# Acute Effects of Low- and High-Speed Resistance Exercise on Cognitive Function in Frail Older Nursing-Home Residents: A Randomized Crossover Study

**DOI:** 10.1155/2021/9912339

**Published:** 2021-08-02

**Authors:** Hélio J. Coelho-Júnior, Samuel da Silva Aguiar, Riccardo Calvani, Anna Picca, Denise de Azevedo Carvalho, Juliana da Costa Zwarg-Sá, Michel Audiffren, Emanuele Marzetti, Marco Carlos Uchida

**Affiliations:** ^1^Laboratory of Applied Kinesiology, School of Physical Education, University of Campinas, Campinas, São Paulo, Brazil; ^2^Rehabilitation Unit, Mãe Mariana Nursing Home, Poá, Brazil; ^3^Università Cattolica Del Sacro Cuore, Rome, Italy; ^4^School of Physical Education, Catholic University of Brasília, Campus I—QS 07—Lote 01—EPCT—Águas Claras—Brasília—DF CEP: 71966-700, Brasília, Brazil; ^5^Physical Education Department, University Center UDF, Brasília-DF, Brazil; ^6^Fondazione Policlinico Universitario “Agostino Gemelli” IRCCS, 00168 Rome, Italy; ^7^Research Centre on Cognition and Learning, UMR CNRS 7295, University of Poitiers, Poitiers, France

## Abstract

**Aim:**

The present study investigated the acute effects of low- and high-speed resistance exercise on the cognitive function of frail older women living in nursing home.

**Materials and Methods:**

Ten institutionalized frail older women were recruited. Rey Auditory Verbal Learning Test and Stroop test were performed before, immediately after, 1 h after, and 24 h after the end of the experimental session. Participants randomly performed low- and high-speed resistance exercise and a control session. Exercise sessions were composed of 4 resistance exercises with 4–8 sets of 4–10 repetitions at moderate intensity.

**Results:**

Results indicated that the performance of Rey Auditory Verbal Learning Test was similarly increased immediately after both low- and high-speed resistance exercises. However, only improvements elicited by low-speed resistance exercise remained significant 1 h after the end of the exercise session. No acute effects of resistance exercise were observed on Stroop performance.

**Conclusion:**

Our findings indicated that both low- and high-speed resistance exercises acutely increased episodic memory in frail older women, whereas no changes on Stroop were observed.

## 1. Introduction

Frailty refers to a reversible state of increased vulnerability to stressful agents, which occurs as a result of multisystem derangements and poor social support [1]. The progression of frailty is marked by increased occurrence of negative events, such as falls and fractures, disability, and loss of independence [1]. Furthermore, a significant association between frailty and cognitive decline has also been documented [2–7].

Findings from cross-sectional studies suggest that cognitive function declines across frailty statuses in nondemented older adults [2–4]. In addition, frail older people have been shown to be at a higher risk of developing dementia compared with nonfrail individuals [5–7]. This scenario is especially concerning, since reduced physical performance and declining cognitive function depict the paradigm of unsuccessfully aging [8] and are therefore recognized as major public health problems [1].

Cognitive impairment [9] and frailty [10] are highly prevalent among nursing-home residents, in whom they contribute to the occurrence of many negative events [11, 12]. Hence, strategies to maintain, or even improve, cognitive function and homeostatic reserves are crucial to foster independence, autonomy, quality of life, and dignity in institutionalized older people [13, 14].

The practice of physical exercise is recommended for nursing-home residents [15] and frail older adults [16]. Among the available exercise training protocols, particular attention has been given to low-speed resistance exercise (LSRE), a training modality in which muscles work to hold or against an applied force at low-to-moderate velocity [17]. LSRE elicits substantial improvements in muscle strength, power, and functional outcomes in older adults with different conditions [18–20] and causes larger increases in the neuromuscular function of frail older people [21].

Notably, some neuromuscular aspects seem to be more dependent on high-speed muscle actions than on those performed at low speed [22, 23]. For this reason, a recent statement from the National Strength and Conditioning Association has recommended including high-speed resistance exercise (HSRE) in exercise programs for older adults [24].

The acute effects of LSRE and HSRE on cognitive function in older adults are still poorly investigated. A recent systematic review and meta-analysis found that LSRE could elicit transitory improvements in global cognitive function, inhibitory control, and cognitive flexibility but not working memory and attention in 447 adults between 20.4 and 72.3 years old [25]. However, these results were not confirmed in subsequent studies [26]. Studies examining older adults are still scarce, but the few available results are encouraging [27, 28]. However, studies have examined “healthy” community-dwelling older adults [25, 27, 28] and, to the best of our knowledge, no trials have investigated the effects of HSRE and/or institutionalized frail older people.

To fill this gap in knowledge, the present study explored and compared the acute effects of LSRE and HSRE on cognitive function in a sample of frail, nondemented older women living in nursing homes.

## 2. Materials and Methods

This was a single-center, randomized crossover study that examined the acute effects of two types of resistance exercise on the cognitive performance of frail older women living in a nursing home. The Research Ethics Committee of the University of Campinas approved the protocol. All study procedures were conducted in compliance with the Declaration of Helsinki and the Resolution 196/96 of the National Health Council. All participants gave their written informed consent prior to participation. This study complies with the criteria of the CONSORT Statement [29].

### 2.1. Participants

Older women were recruited by convenience between August and December 2019 from a private nursing home located in the eastern region of São Paulo State, southern Brazil. Nursing-home residents were considered to be eligible for inclusion if they satisfied the following criteria: (a) ≥60 years of age; (b) frail according to Fried's criteria [30]; (c) possessed sufficient physical and cognitive abilities to perform all measurements required by the protocol; and (d) received physician approval to participate. Candidates were excluded if they had participated in a structured physical exercise program in the previous six months, had uncorrected visual deficit or color blindness, were prescribed with hormone replacement therapy and/or psychotropic drugs, had experienced any cardiovascular event (e.g., myocardial infarction) or complication in the past six months, or suffered from dementia according to Mini Mental State Examination (MMSE) scores adjusted by education level [31].

Participants performed three experimental sessions: LSRE, HSRE, or control session (CS), each separated by seven days. The order of experimental sessions was defined randomly using a computer-generated list of random numbers based on a 1 : 1 : 1 ratio. All experiments were performed in the rehabilitation unit of the nursing home. Food consumption was maintained constant during the previous 48 h and participants consumed a standard breakfast 60–90 min before the beginning of the experimental session.

### 2.2. Ten-Repetition Maximum (10RM) Test

Participants were familiarized with resistance exercises prior to a 10-repetition maximum (10RM) test. 10RM tests were performed for the three following exercises: squat on the chair (until 90° knee flexion), seated unilateral hip flexion, and seated unilateral knee extension. Before the tests, participants performed a brief specific warm-up using light loads. Afterward, the 10RM load was determined in up to five attempts, with a 3 min interval among trials. The resistance was increased according to participants' capacity to perform more than one successful repetition maximum with the proper move and full range of motion. The test was completed when participants were unable to perform more than 10 repetitions using proper technique [32]. Subsequently, the 1-repetition maximum (1RM) was calculated according to the following formula [33]:

(1) $$\begin{array}{c} 1\text{RM}=\frac{10\text{RM}}{\left[1.0278\left(0.0278\times 10\right)\right]}. \end{array}$$

### 2.3. Experimental Sessions

Exercise sessions were performed in the mornings (07:30 am–10:00 am) under the supervision of at least two fitness instructors. After a brief warm-up, participants performed the following exercises using an adjustable weight vest and ankle weights (DOMYOS®, Shanghai, China): (1^st^) squat on the chair (until 90° knee flexion), (2^nd^) seated unilateral hip flexion, (3^rd^) seated unilateral knee extension, and (4^th^) bilateral calf raise. The total volume (sets × repetitions × load = ∼800 kg) was equalized among exercise sessions.

LSRE and HSRE were designed according to the peculiarities of each type of resistance exercise [34]. During LSRE, participants performed 4 sets of 8–10 repetitions at 70–75% of 1RM. The concentric and eccentric phases were carried out for 2 s. For HSRE, exercises were performed 8 times (sets) with 3–5 repetitions at 70–75% of 1RM. The concentric phase was performed as fast as possible, and the eccentric phase was carried out for 2 s. Bilateral calf raise was performed with the load of unilateral knee extension. A researcher was responsible for monitoring and ensuring that the velocity of muscle contraction was performed according to the protocol. Particularly, verbal encouragement was provided in the HSRE. During CS, participants remained seated on a comfortable chair for approximately 30 min.

### 2.4. Cognitive Function

All cognitive tests were performed before (rest), immediately after (IA), 1 h after, and 24 h after the end of the exercise session. Cognitive assessments were conducted face to face by a trained researcher in a private silent room. Shorter and simplified versions of cognitive tests were used to familiarize participants before actual testing.

#### 2.4.1. Rey Auditory Verbal Learning Test (RAVLT)

RAVLT is a neuropsychological tool widely used for testing episodic memory [35]. The test consists in read aloud two lists (*A* and *B*) of 15 substantives each with a 1 s interval between each two words. At the beginning of the test, list *A* was read five consecutive times by a researcher. Then, participants were requested to recall as many words as possible after each trial (*A*1–*A*5). List *B* (interference list) with 15 new substantives was read after *A*5 and words had to be retrieved (*B*1). Finally, participants were asked to recall the words from list *A* immediately after the interference list (*A*6, immediate recall) and following a delay of 20 min (*A*7, delayed recall), without listening to list *A* again [35]. Eight different lists (for lists *A* and for lists *B*) with similar frequency of words of the same semantic category were presented at each session to avoid learning effects. Five summary scores were used to assess episodic memory, delayed memory, verbal learning, and susceptibility to interference [35]:

(2) $$\begin{array}{c} \text{Verbal learning }\left(\text{VL}\right)\text{ curve}=A1,A2,A3,A4,A5,\\ \text{Verbal learning }\left(\text{VL}\right)\text{ score}=\sum A1-A5-\left(5*A1\right),\\ \text{Forgetting speed }\left(\text{FS}\right)=A7-A6,\\ \text{Immediate recall }\left(\text{IR}\right)=\text{The sum of correct wordsretrieved in }A6,\\ \text{Delayed recall }\left(\text{DR}\right)=\text{The sum of correct words retrieved in }A7. \end{array}$$

#### 2.4.2. Stroop Test

A computerized version of the Stroop test (TESTINPACS™) [36] was used to measure the reaction time (ms) and the number of correct responses for each stimulus (control, congruent, and incongruent) [36]. For the test, participants remained seated in front of a 17ʺ color monitor. The distance from the monitor was set according to participant vision needs. The Stroop test involves three experimental conditions. In the control condition, the stimulus was a rectangle painted in green, yellow, blue, or red. Two possible responses, corresponding or not to the color of the rectangle, were shown at the lower corners of the monitor, and participants were requested to tell the color corresponding to the rectangle. In the congruent condition, the stimulus was the name of a color (e.g., red written in white). Two possible responses (i.e., name of two colors written in white, one corresponding to the stimulus and the other not) were presented at the lower corners of the monitor and participants had to read the color word. In the incongruent condition, the stimulus is a color name exhibited in a nonmatching color (e.g., red written in blue). Two possible responses, the name of the color word (red in the example) and the color of the ink (blue in the example), were presented at the lower corners of the monitor. Participants were requested to name the color of the ink and inhibit the reading of the word. A total of 36 stimuli (12 trials for each condition) were randomly provided, and reaction time was recorded in ms. After the participant vocal response, a researcher was responsible for immediately pressing the corresponding key (← or ⟶). This protocol was established after a pilot study in which we observed that participants of the present study took too long or were unable to return the hand to the initial position, if they had to take it off, even if the keyboard was composed by only two keys.

### 2.5. Statistical Analysis

Normality of data was tested using the Kolmogorov-Smirnov test. Intragroup and intergroup comparisons at different timepoints for RAVLT and Stroop variables were performed using two-way analysis of variance (ANOVA) followed by Dunnett's post hoc test. Greenhouse-Geisser corrections were applied for data that violated sphericity assumptions. The level of significance was set at 5% (*p* < 0.05), and all analyses were run using GraphPad Prism software (GraphPad Software, Inc., San Diego, CA, USA).

## 3. Results

Eighteen nursing-home residents were recruited for the present study and fifteen accepted to be evaluated for inclusion. Of these, four had dementia according to MMSE scores and one left the study after the 10-RM test, leaving a total of 10 older women. The main characteristics of the study sample are shown in Table 1.

**Figure 1:**
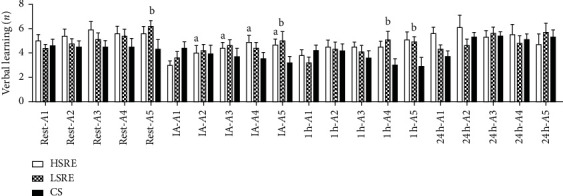
Point-by-point analysis of Rey Auditory Verbal Learning Test (RAVLT). CS = control session; LSRE = low-speed resistance exercise; HSRE = high-speed resistance exercise. ^a^*p* < 0.05 versus *A1* in HSRE; ^b^*p* < 0.05 versus *A*1 in LSRE.

**Figure 2:**
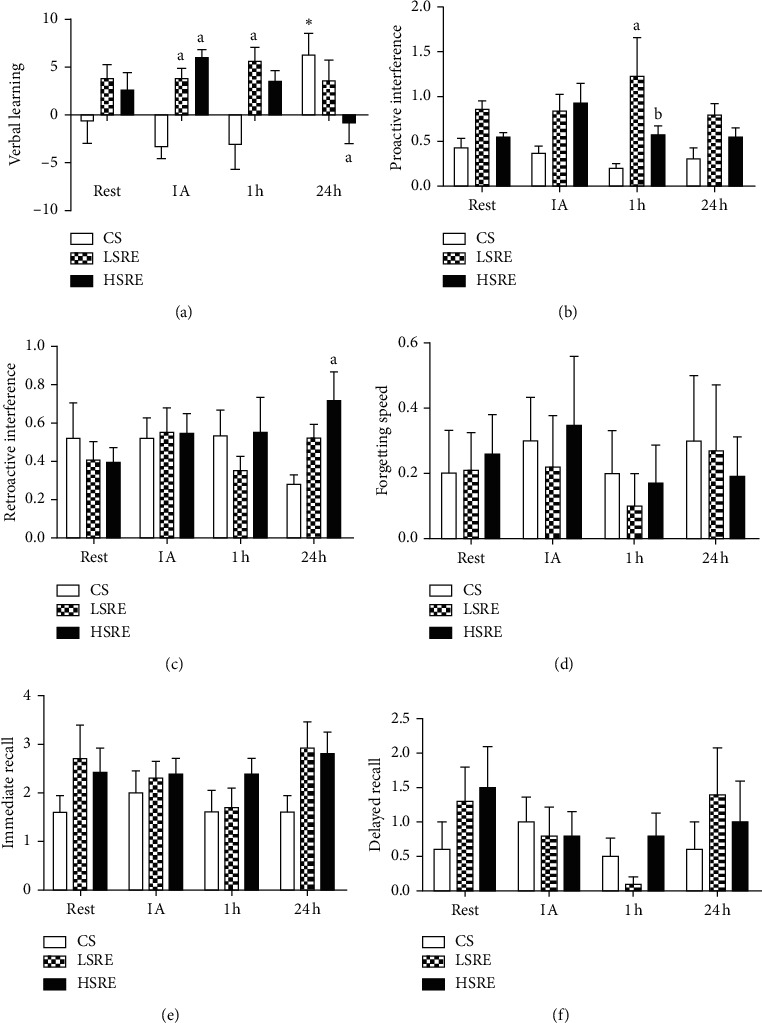
Rey Auditory Verbal Learning Test (RAVLT) scores at different timepoints according to group allocation. CS = control session; LSRE = low-speed resistance exercise; HSRE = high-speed resistance exercise; IA = immediately after. ^*∗*^*p* < 0.05 versus rest; ^a^*p* < 0.05 versus CS.

The acute effects of resistance exercise on RAVLT are shown in Figures 1 and 2. Significant time and interaction effects were observed. The point-by-point analysis indicated that VL increased linearly IA from *A1* to *A5* in HSRE (Figure 1; *p* < 0.001). A significantly higher *A5* was observed at rest (*p*=0.01), IA (*p*=0.001), and 1 h after exercise (*p*=0.001) in LSRE. *A4* was only improved 1 h after LSRE (*p* < 0.001; Figure 1).  Figure 2(a) shows overall VL scores. A significant interaction effect was observed. Higher VL scores were observed IA in both HSRE (*p*=0.001) and LSRE (*p*=0.01) compared with CS but remained greater at 1 h only in LSRE (*p*=0.01). At 24 h, VL was significantly higher in CS relative to baseline (*p*=0.001) and HSRE (*p*=0.01). No changes were observed in FS, IR, and DR in response to any session and no other between-group differences were found.

**Figure 3:**
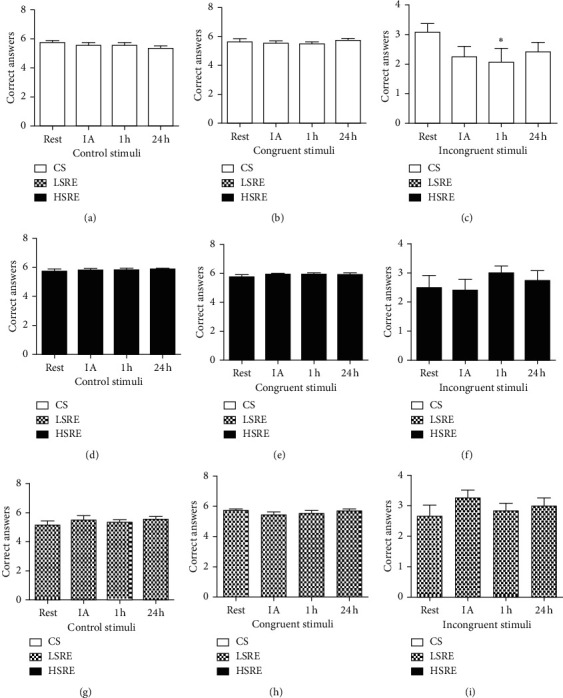
Correct answers in the Stroop test. CS = control session; LSRE = low-speed resistance exercise; HSRE = high-speed resistance exercise; IA = immediately after. ^*∗*^*p* < 0.05 versus rest.

**Figure 4:**
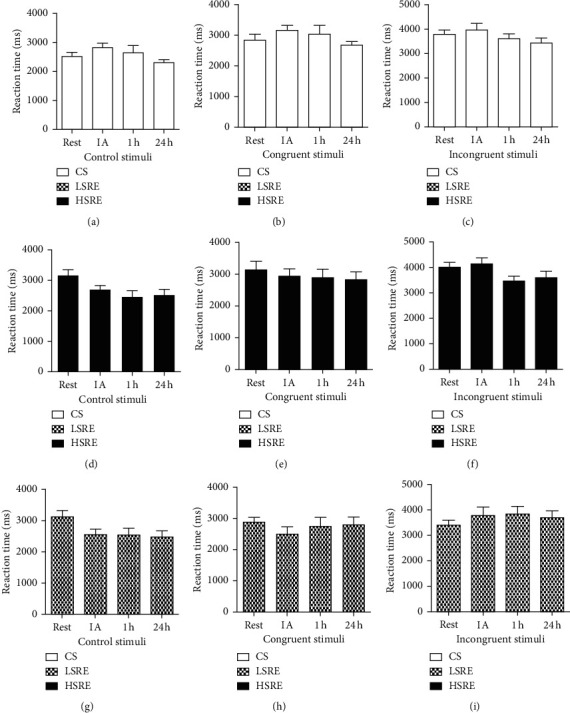
Reaction time in the Stroop test. CS = control session; LSRE = low-speed resistance exercise; HSRE = high-speed resistance exercise; IA = immediately after.

The acute effects of resistance exercise on Stroop performance are shown in Figures 3 and 4. Neither LSRE nor HSRE affected the number of correct answers (Figures 3(b) and 3(c)), which was significantly reduced at 1 h during the incongruent stimulus in CS (Figure 3(a)). No changes were observed in reaction time (Figure 4) and no between-group differences were observed.

## 4. Discussion

The findings of the present study indicate that, in frail older women living in nursing home, both LSRE and HSRE acutely increased VL, an indicator of episodic memory. Different patterns of cognitive changes were observed among experimental conditions, so that VL remained higher during 1 h following LSRE, while it was only increased IA HSRE. Notably, VL was significantly reduced in HSRE 24 h after the exercise session. No acute effects of exercise were observed on Stroop performance, but the number of correct answers was significantly lower in CS during the incongruent stimulus.

Acute effects of resistance exercise on cognitive function in older adults have been sparsely investigated. Furthermore, the few available studies were conducted on “healthy” community-dwelling older adults [25, 27, 28]. Hsieh et al. [27] found improved working memory 10 min following an LSRE session in nondemented older men. Similarly, Naderi et al. [28] observed improved working memory in older adults who performed LSRE at 40% and 70% 1RM. In a recent systematic review and meta-analysis, Wilke et al. [25] reported that an acute session of resistance exercise could improve inhibitory control and cognitive flexibility but not working memory in healthy adults.

Our results provide a prima facie case for the differential effects of LSRE and HSRE on verbal memory in frail older women. Notably, LSRE elicited longer learning improvements in comparison to HSRE, even if results seem to be greater IA HSRE compared to LSRE. The possible mechanisms underlying such effects were not investigated in the current study. The total exercise volume was equalized among groups, thereby allowing discarding the hypothesis that our findings could be explained by different workloads.

On the other hand, differences in the duration of muscle contraction between training modalities could offer a plausible explanation for our results, given that muscle contractions occurred at low velocity during LSRE and at high velocity during HSRE. Indeed, muscle contraction velocity might affect neuroendocrine responses to exercise [37]. For instance, insulin-like growth factor-1 (IGF-1) is acutely increased in response to LSRE in older adults [38] and systemic IGF-1 levels are significantly associated with verbal memory and hippocampal perfusion and volume [39]. In addition, acute increases in IGF-1 levels have been observed in brain areas associated with memory formation (e.g., hippocampus and cortex) [40], which seems to be critically involved in exercise-induced improvements in neuronal activation and cell proliferation [41].

Serum testosterone, sex hormone binding globulin (SHBG), and cortisol levels tend to be lower IA HSRE in comparison to LSRE with an equivalent total volume [37], suggesting that other mechanisms may be associated with acute HSRE-induced transitory cognitive gains. Nitric oxide (NO) is a possible candidate to explain improved verbal memory after HSRE [42]. Indeed, infusion of the NO precursor l-arginine ameliorated learning and memory and increased the length of cortical capillaries in rats [43]. These benefits were abrogated by the administration of the NO synthase inhibitor N^G^-nitro-l-arginine methyl ester (l-NAME) [43]. Remarkably, NO concentrations were found to be increased in saliva after an acute session of HSRE in older women [42]. However, these speculations cover only few of the myriad of mechanisms that can be responsible for the acute effects of resistance exercise on cognition and future studies are required to better explore the matter.

Noticeably, VL performance was significantly reduced at 24 h in HSRE. These results go against the inverted-U shaped curve proposed by Audiffren [44], in which low- and high-intensity exercise sessions are expected to induce small transitory cognitive improvements, while greater changes may be observed after moderate-intensity exercise. One possible explanation for this phenomenon may be that frail older adults have high baseline cortisol levels [45]. Although HSRE was performed at moderate intensity, frail older women may have reduced resiliency to physical stress [46], resulting in exaggerated hypercortisolemia during recovery with consequent transient deterioration of memory performance [47].

Another possible explanation resides in the fact that explosive muscle contractions require more attention than those performed at lower speed [48]. Accordingly, sustained attention during HSRE might lead to a transient state of mental fatigue, thereby reducing motivation [49] and cognitive performance [50]. This can be particularly true in frail older people [51] and in nursing-home residents [52], whose homeostatic reserve is typically reduced.

The effects of resistance exercise on Stroop performance remain equivocal. In keeping with our findings, numerous studies did not report improvements in Stroop performance following LSRE in middle-aged and older adults with mild cognitive impairment [53]. On the other hand, Johnson et al. [54] found increased performance on the incongruent stimulus up to 1 h after resistance exercise. These findings are supported by a recent systematic review and meta-analysis showing that a single LSRE session may induce moderate improvements on inhibitory control [25].

The above-mentioned findings are hard to reconcile. A possible explanation for the divergent results may be that Johnson et al. [54] used a circuit-based resistance exercise, which has a high aerobic component [55]. Differences in age and health status of participants across studies may offer an additional explanation for these discrepancies. Indeed, healthy adults were investigated by Johnson et al. [54] and Wilke et al. [25], while frail older women were enrolled in our study.

Our findings may have important practical implications. Acute hemodynamic responses to resistance exercise have been shown to predict long-term cardiovascular adaptations [56] and contribute to lower cardiac risk during the activities of daily living [57]. There exists the possibility that the same scenario might occur in relation to cognitive function. Hence, acute cognitive responses to resistance exercise could serve as a metric to identify responders and nonresponders [58]. This information, in turn, may be used to design personalized training programs as well as to interpret results of clinical trials testing long-term LSRE and HSRE protocols in frail older adults.

Of particular interest is the reduced RAVLT performance observed 24 h after HSRE. This finding suggests that the frail older people should be closely monitored after performing HSRE, as they may develop memory deficits following training. Such effects are transitory and should return to regular levels within 48 hours. However, health professionals responsible for exercise prescription must be aware of this scenario and be on the lookout for signs of mental fatigue. Hence, rest intervals longer than 24 h might be necessary after HSRE for avoiding cognitive overloading.

Our analysis also revealed two unexpected results. Notably, *A*5 was significantly increased at rest in LSRE but not in the other two situations, whereas exclusive increases in VL were observed 24 hours after the CS. These observations might be attributed to within-person fluctuations in frailty status. In fact, Stolz et al. [59] noted that frail very old women with low educational levels were more susceptible to show unstable health, characterized according to frequent oscillations in a multidomain frailty index.

Another possible explanation for our results is the presence of learning effect, despite our attempts to reduce it using randomization and eight different RAVLT lists. According to Scharfen et al. [60], retest effects in memory capacity tests increased up to the fourth test administration until they reached a plateau and are highly influenced by the length of the test-retest interval. Hence, future studies proving larger intervals between sessions are still necessary to confirm our results.

Our work, although reporting novel findings, is not free of limitations. As mentioned earlier, the study was not designed to explore the mechanisms underlying acute effects of exercise on cognition. Furthermore, even though participants were thoroughly characterized, the study sample was small. Therefore, our results need to be confirmed by larger-scale studies. Along similar lines, findings were obtained in frail older women living in nursing home and may not be generalized to noninstitutionalized robust older adults or to the male gender. Finally, the reaction time in the Stroop test was dependent on the researcher's velocity response to participant stimulus. However, the same investigator conducted all experiments to limit interobserver variability.

## 5. Conclusion

Our findings indicate that LSRE and HSRE acutely increased VL, an indicative of episodic memory, in frail older women living in nursing home. However, VL remained improved only during 1 h after LSRE. Remarkably, the performance on the RAVLT was reduced 24 h after HSRE. While no mechanistic explanation may be provided for this finding, it might be hypothesized that the sustained attention required by HSRE could induce a transient state of mental fatigue, resulting in reduced cognitive performance. Therefore, frail older persons engaged in HSRE programs may need rest intervals longer than 24 h to avoid cognitive overload. Finally, we did not observe any acute effects of either LSRE or HSRE on Stroop test. Yet, the number of correct answers was lower in CS during the incongruent stimulus.

## Figures and Tables

**Table 1:** Main characteristics of study participants.

Variables	*n* = 10
Age, years	86.2 ± 10.2
BMI, kg/m^2^	23.5 ± 1.3
Period of institutionalization, years	1.0 ± 0.0
MMSE, points	16.4 ± 4.4

*Comorbidities (n)*	
Hypertension	8
Osteoarthritis	6
Stroke	2
Diabetes	2

Data are presented as mean ± SD. BMI = body mass index; MMSE  = Mini Mental State Examination.

## Data Availability

The data are available from the first author upon request.
